# Validity of a Self-administered Food Frequency Questionnaire for the Estimation of Acrylamide Intake in the Japanese Population: The JPHC FFQ Validation Study

**DOI:** 10.2188/jea.JE20170186

**Published:** 2018-12-05

**Authors:** Ayaka Kotemori, Junko Ishihara, Misako Nakadate, Norie Sawada, Motoki Iwasaki, Tomotaka Sobue, Shoichiro Tsugane

**Affiliations:** 1 Epidemiology and Prevention Group, Center for Public Health Sciences, National Cancer Center, Tokyo, Japan; 2 Department of Food and Life Science, Azabu University, Kanagawa, Japan; 3 Division of Nutrition Science, Graduate School of Sagami Women’s University, Kanagawa, Japan; 4 Department of Environmental Medicine and Population Sciences, Graduate School of Medicine, Osaka University, Osaka, Japan

**Keywords:** acrylamide, validity, food frequency questionnaire, dietary record, cooking method

## Abstract

**Background:**

Acrylamide, a probable carcinogen to humans, forms during high temperature cooking. Dietary exposure to acrylamide among the Japanese population is unknown. We aimed to establish and validate a method to assess acrylamide exposure among the Japanese population using a food frequency questionnaire (FFQ) from the Japan Public Health Center-based prospective study.

**Methods:**

Validation studies for the FFQ were conducted in 1994 (Cohort I, *n* = 215) and 1996 (Cohort II, *n* = 350). The 28-day dietary records (DRs) were collected over 1 year. The FFQ was distributed before and after DR collection. Data for acrylamide exposure were based on reported measurements in Japan, and calculations considered the cooking process for specific vegetables in a home setting. Spearman’s rank correlation and weighted kappa coefficients were calculated from energy-adjusted data.

**Results:**

Mean acrylamide intake levels estimated from DRs for Cohorts I and II were 6.78 (standard deviation [SD], 3.89) µg/day and 7.25 (SD, 3.33) µg/day, and corresponding levels estimated from the FFQ were 7.03 (SD, 4.30) µg/day and 7.14 (SD, 3.38) µg/day, respectively. Deattenuated correlation coefficients for men and women were 0.54 and 0.48 in Cohort I and 0.40 and 0.37 in Cohort II, respectively. Weighted kappa coefficients were over 0.80 in all cases. The main contributing food groups from DRs were beverages, confectioneries, vegetables, potatoes and starches, and cereals.

**Conclusions:**

High kappa values validate the use of FFQ in epidemiological studies. The marked contribution of cooked vegetables indicates the importance of considering household cooking methods in assessing acrylamide intake levels in the Japanese population.

## INTRODUCTION

Acrylamide is used in the production of industrial products, such as adhesives, and is known to be contained in tobacco smoke.^1^ The International Agency for Research on Cancer classified acrylamide as a probable carcinogen in humans (Group 2A) in 1994.^2^ Subsequently, acrylamide was found in highly heated foods, such as french fries, for the first time by Swedish scientists in 2002.^3^ Therefore, diet is also a considerable source of acrylamide exposure in the general population.

To clarify the risk of dietary acrylamide intake on carcinogenicity in humans, epidemiological studies mainly use food frequency questionnaires (FFQs) to assess acrylamide intake. Since FFQs can be used to rank participants according to levels of intake, associations, such as those between food intake and risk of disease, can be examined. However, there has been no study establishing an acrylamide database and validating acrylamide intake estimates from FFQs in the Japanese population.

Foods contributing to acrylamide intake are expected to differ between Asian and western populations. The main foods contributing to acrylamide intake among western people are potato-based foods, wheat-based products, and coffee.^4^^–^^6^ However, in a report of acrylamide content in foods commonly consumed by Japanese people, foods associated with household cooking, such as stir-fried vegetables, also contained acrylamide.^7^ Therefore, the cooking method of foods prepared at home is an important consideration in the estimation of dietary acrylamide intake levels.

We oversee the Japan Public Health Center-based (JPHC) Study, a prospective study of a large Japanese cohort that was launched in the 1990s and continues to be followed today. The present study aimed to establish a database of acrylamide-containing foods commonly consumed by Japanese people and to validate acrylamide intake from a self-administered dietary questionnaire compared to a 28-day weighted dietary record (DR) in the JPHC Study.

## METHODS

### Data collection and study participants

Details of the study design and participants’ characteristics were described elsewhere.^8^^–^^10^ In brief, validation studies for FFQ were conducted among subsamples from two cohorts: Cohort I from February 1994 and Cohort II from May 1996. In the Cohort I area, 215 participants completed 28-day (14-day for participants in Okinawa) DRs and the FFQ, and 209 participants completed DRs and two FFQs. In the Cohort II area, 350 participants completed DRs and the FFQ, and 289 participants completed DRs and two FFQs. Cohort I and Cohort II were independently conducted studies that collected dietary records among different populations. This study did not undergo ethics approval, since it was conducted before the administration of ethics guidelines for epidemiological research in Japan, which mandate such approval. However, oral or written informed consent was obtained from the participants before the start of the study. Further, for statistical analysis, our study protocol was approved by the ethics committee of Osaka University (approved number 15131).

### Development of a database of acrylamide-containing foods

A total of 10 reports^7^^,^^11^^–^^19^ were available through July 2016, and we selected the measurements in these reports according to the following criteria: 1) sample size of measurements was two or more; 2) mean or median value of measurements was reported or able to be calculated from reported data; 3) if there were several reports for the same food, values measured in a Japanese sample and from larger sample sizes were preferentially used; 4) if both mean and median were reported, median was preferentially used; and 5) if the measurements were below quantitative or qualitative limits, values equating to half that of the quantitative or qualitative concentrations were used.

To calculate acrylamide intake from DRs, we used the Standard Tables of Food Composition in Japan, Fifth Revised and Enlarged Edition (5th FCT), which contains 1,878 food items. Since acrylamide measurement values in existing literature are limited, we attempted to organize relevant acrylamide-containing items of the 5th FCT into more discrete categories for which acrylamide measurements are available. We categorized the 1,878 food items from the 5th FCT using the following criteria: a) same or similar species, same or similar parts, and same or similar cooking methods as an acrylamide-containing food with available data; b) when a food consisted of several foods containing acrylamide (eg, confectioneries), the acrylamide values were calculated using measurements from the database for each food item; further, c) oils and fats, fresh food, non-processed food, and non-heated food were assigned a value of zero; and d) other foods that did not meet the above criteria were treated as missing values. After applying these acrylamide criteria to the 5th FCT, 282 (15%) out of 1,878 food items from the 5th FCT were designated as acrylamide-containing foods. The number of foods treated as non-acrylamide-containing foods (criteria c) was 1,276, and the number of foods treated as missing (criteria d) was 320.

We did not apply available acrylamide concentration data for mixed dishes, which were not listed in the 5th FCT (eg, stir-fried vegetables and meats, fried fish with batter, and curry), because cooked foods listed in the 5th FCT were limited and both DR and FFQ were conducted using food-based methods. However, acrylamide concentration varies depending on the cooking method, even for the same food.^7^ Therefore, in addition to the food items from the 5th FCT, the following foods were also used to calculate acrylamide intake from cooked foods: potatoes (deep-fried, baked, stir-fried, and stir-fried lightly as preparation), onions (deep-fried, baked, stir-fried, and stir-fried lightly as preparation), bean sprouts (deep-fried, baked, and stir-fried), asparagus (deep-fried, baked, and stir-fried), sweet peppers (deep-fried, baked, and stir-fried), squash (deep-fried, baked, and stir-fried), cabbage (deep-fried, baked, and stir-fried), string beans (deep-fried, baked, and stir-fried), eggplant (deep-fried, baked, and stir-fried), broccoli (deep-fried, baked, and stir-fried), podded peas (deep-fried, baked, and stir-fried), sweet potato (deep-fried), toasted bread, deep-fried batter, and stir-fried rice.^7^ Ultimately, we expanded the food items listed in the 5th FCT from 1,878 to 1,917, and 321 food items (17%) were designated as acrylamide-containing foods.

### Calculation of dietary acrylamide intake from DRs

Participants recorded menus, food and beverage names, and the amount consumed (weighed using a scale) in the specific food diary. Dieticians checked the records and coded each food using the food item code of the 5th FCT. To calculate acrylamide intake from specific cooked foods not listed in the 5th FCT, dieticians used the menu to determine the cooking methods of these foods. Nine cooking methods (raw, boiled, deep-fried, deep-fried with batter, baked, stir-fried, steamed, stir-fried lightly as preparation, and unclear) were coded. Dietary acrylamide was calculated by multiplying the concentration and amount consumed for each food and summing these values by day. Energy intake was also calculated using the 5th FCT.

### Calculation of dietary acrylamide intake from FFQs

Our FFQ was designed to assess habitual dietary intake for the previous 1 year.^9^^,^^10^ Out of 147 food items, the following 28 (19%) were designated as acrylamide-containing foods: rice, miso, beer, baked fish paste, bread, rice cake, Japanese-style confectioneries, cakes, biscuits and cookies, chocolates, peanuts, fried tofu, sencha (a type of green tea), bancha (a type of green tea), oolong tea, black tea, coffee, canned coffee, soup, potatoes, sweet potato, onions, bean sprouts, sweet peppers, squash, cabbage, snap beans, and broccoli. For rice, bread, potato, sweet potato, and vegetables (onions, bean sprouts, sweet peppers, squash, cabbage, snap beans, and broccoli), we calculated acrylamide intake by considering the cooking methods used because our original FFQ only estimated the amount of raw food intake. Weighted average values of acrylamide content for these foods were calculated using the proportion of each cooking method, obtained from the DRs of Cohort I and Cohort II separately. We inserted the proportions of cooking method calculated from Cohort I in the calculation of acrylamide intake in Cohort II and vice versa. Acrylamide intake from each food was calculated by multiplying the concentration of acrylamide for each food by the eating frequency and portion size.

We also estimated acrylamide intake from fried batter. Our original FFQ contained a question inquiring about eating frequency for fried foods with batter: “How often do you consume deep fried foods with batter?” Respondents chose their response from six frequency categories: almost never, 1–3 times/month, 1–2 times/week, 3–4 times/week, 5–6 times/week, and daily. Acrylamide intake from fried batter was estimated by multiplying the eating frequency for fried foods with batter with the amount of acrylamide intake from fried batter per day calculated from the DRs. Total daily acrylamide intake from the FFQ was calculated by summing the acrylamide intake for each food item and fried batter.

### Statistical analysis

Mean dietary acrylamide intake was calculated by sex and cohort. Percentage differences of acrylamide intake between DR and FFQ were calculated using the following formula: (acrylamide intake from FFQ − acrylamide intake from DR)/acrylamide intake from DR × 100. Spearman’s rank correlation coefficients (CC) between DRs and the FFQ were calculated for crude and energy-adjusted values. Energy adjustment was conducted using the residual method. In addition, de-attenuated correlation coefficients were also calculated because the CC between DRs and the FFQ are attenuated by individual variations in daily intake. De-attenuation was calculated using the following formula: de-attenuated CC = observed energy-adjusted CC × $\sqrt{1+(\lambda /n)}$, where *λ* is the ratio of intra- to inter-individual variation of DRs, and *n* is the number of DRs for each participant (28 days). For cross-classification analysis, energy-adjusted acrylamide intake from DRs and the FFQ were divided into five categories by quintile, and the percentage of participants among the same, adjacent, and opposite categories were calculated using both quintile numbers. Moreover, weighted kappa coefficients were calculated. Contribution of each food to total acrylamide intake were calculated as the percentage of acrylamide intake from each food to the total amount of acrylamide intake from DR data. For sensitivity analysis, we conducted the same analysis but excluded acrylamide intake from fried batter. All analyses were performed using SAS (version 9.3, SAS Institute Inc., Cary, NC, USA).

## RESULTS

### Validity and reproducibility of the FFQ

Mean acrylamide intake from DRs was about 7 µg/day, and the 95th percentile value was 0.23 µg/kg body weight/day (Table 1). About 4% overestimation was observed in Cohort I, and a bare underestimation was observed in Cohort II.

**Table 1.** Comparison of mean acrylamide intake from DR and FFQ

	DR				FFQ_V				%^a^
	
	Mean	(SD)	Median	(5th %-ile, 95th %-ile)	Mean	(SD)	Median	(5th %-ile, 95th %-ile)	
**Cohort I**									
*Crude acrylamide intake (µg/day)*									
Men (*n* = 102)	6.72	(3.70)	5.84	(2.94, 13.43)	6.74	(3.96)	6.11	(2.07, 13.10)	0
Women (*n* = 113)	6.84	(4.07)	6.02	(3.11, 13.15)	7.30	(4.59)	6.36	(2.30, 14.16)	7
All (*n* = 215)	6.78	(3.89)	5.92	(2.98, 13.43)	7.03	(4.30)	6.13	(2.16, 13.74)	4
*Crude acrylamide intake (µg/body weight/day)*									
Men (*n* = 102)	0.10	(0.06)	0.09	(0.04, 0.20)	0.11	(0.07)	0.09	(0.03, 0.20)	5
Women (*n* = 113)	0.13	(0.08)	0.11	(0.05, 0.24)	0.14	(0.09)	0.12	(0.04, 0.28)	5
All (*n* = 215)	0.12	(0.07)	0.10	(0.05, 0.23)	0.12	(0.08)	0.10	(0.03, 0.25)	2
**Cohort II**									
*Crude acrylamide intake (µg/day)*									
Men (*n* = 174)	7.53	(3.60)	6.89	(3.41, 14.80)	6.94	(3.42)	6.37	(2.13, 13.67)	−8
Women (*n* = 176)	6.97	(3.04)	6.39	(3.16, 12.83)	7.34	(3.34)	6.60	(2.63, 13.51)	5
All (*n* = 350)	7.25	(3.33)	6.61	(3.34, 14.07)	7.14	(3.38)	6.49	(2.43, 13.64)	−2
*Crude acrylamide intake (µg/body weight/day)*									
Men (*n* = 174)	0.12	(0.06)	0.11	(0.05, 0.23)	0.11	(0.06)	0.10	(0.03, 0.22)	−7
Women (*n* = 176)	0.13	(0.06)	0.12	(0.06, 0.23)	0.14	(0.06)	0.13	(0.05, 0.26)	5
All (*n* = 350)	0.12	(0.06)	0.11	(0.06, 0.23)	0.12	(0.06)	0.12	(0.04, 0.25)	3

DR, dietary record; FFQ_V, food frequency questionnaire for validation analysis; SD, standard deviation.

^a^Percentage differences (%) were calculated from following formula: (“mean FFQ_V” − “mean DR”)/“mean DR” × 100.

Ranges of energy-adjusted CCs for Cohorts I and II were 0.34 to 0.48 and deattenuated CCs were 0.37 to 0.54 (Table 2). From cross-classification analysis, 26–31% of participants were assigned to the ‘same’ category, 63–74% were assigned to the ‘same and adjacent’ category, and 2–4% were assigned to the ‘extreme’ category. The weighted kappa coefficients were over 0.80. For correlations between FFQs for reproducibility, the range of energy-adjusted CCs were 0.56 to 0.62 for Cohorts I and II.

**Table 2.** Evaluation of the relationship between DR and FFQ_V and between FFQs by correlation and cross-classification analysis

	Validity^a^			Cross-classification^b^				Reproducibility^c^	
		
	Crude	Energy-adjusted	Deattenuated^d^	Same category	Same and adjacent category	Extreme category	Weighted κ coefficient	Crude	Energy-adjusted
**Cohort I**									
Men (*n* = 102)	0.36	0.48	0.54	31	74	3	0.86	0.64	0.62
Women (*n* = 113)	0.30	0.42	0.48	27	70	4	0.85	0.62	0.56
**Cohort II**									
Men (*n* = 174)	0.29	0.37	0.40	30	65	3	0.84	0.65	0.61
Women (*n* = 176)	0.23	0.34	0.37	26	63	2	0.83	0.67	0.58

DR, dietary record; FFQ_V, food frequency questionnaire for validation analysis.

^a^Spearman’s correlation coefficients between DRs and the FFQ_V.

^b^Percentages were presented based on the cross-classification by quintile between DRs and the FFQ_V.

^c^Spearman’s correlation coefficients between two FFQs.

^d^Deattenuated CC = Energy-adjusted CC × $\sqrt{1+(\lambda /n)}$, where *λ* is the ratio of within- to between-individual variance and *n* is number of DRs.

In the sensitivity analysis, where acrylamide intake from fried batter was excluded, the Spearman’s CCs for crude and energy-adjusted intake were unchanged: Cohort I, *r* = 0.37 and *r* = 0.48 in men and *r* = 0.31 and *r* = 0.43 in women; Cohort II, *r* = 0.29 and *r* = 0.37 in men and *r* = 0.23 and *r* = 0.33 in women, respectively.

### Contribution of each food group to total acrylamide intake from DRs in Japanese people

Beverages, confectioneries, vegetables, potatoes and starches, and cereals contributed to the total acrylamide intake (Table 3). The top five food groups accounted for about 90% of total acrylamide intake. Among all foods, acrylamide from potatoes contributed the most, followed by green teas, and coffees and cocoas. When considering the cooking method of vegetables, sweet peppers, bean sprouts, and onions were contributing foods.

**Table 3.** Contribution to total acrylamide intake by food groups from DRs

Food group number	Food group name	Proportion (%)	Number of assigned foods	Top 5 contributing foods (%)									

				1		2		3		4		5	
16	Beverages	32.1	22	Green teas	(13.8)	Coffees and cocoas	(13.2)	Fermented alcoholic beverages	(2.9)	Fermented teas	(1.2)	Mugi-cha	(1.0)
15	Confectioneries	17.8	103	Traditional dry confectioneries	(5.8)	Biscuits and cookies	(5.5)	Traditional fresh and semi-dry confectioneries	(2.0)	Cakes, buns and pastries	(1.7)	Chocolates	(1.4)
6	Vegetables	17.6	5	Sweet peppers	(4.9)	Bean sprouts	(4.5)	Onions	(3.3)	Cabbages	(1.6)	Eggplants	(1.4)
2	Potatoes and Starches	14.8	2	Potatoes	(14.0)	Sweet potatoes	(0.9)						
1	Cereals	7.9	51	Rice	(4.4)	Noodles dried by frying	(1.7)	Breads	(1.5)	Bread crumbs	(0.4)		
5	Nuts and Seeds	3.7	16	Sesame seeds	(2.3)	Peanuts	(0.8)	Walnuts	(0.4)	Almonds	(0.1)		
17	Seasonings and Spices	3.2	14	Roux	(1.7)	Miso	(0.8)	Soy sauces	(0.6)				
4	Pulses	1.0	18	Tofu (baked or fried)	(0.6)	Roasted and ground soy beans	(0.3)						
3	Sugars and Sweeteners	0.8	6	Brown sugar	(0.8)								
7	Fruits	0.6	18	Dried fruits	(0.6)								
10	Fishes and Shellfishes	0.5	4	Fish paste products (baked or fried)	(0.5)								
8	Mushrooms	0	0										
9	Algae	0	0										
11	Meats	0	0										
12	Eggs	0	0										
13	Milks	0	0										
14	Fats and Oils	0	0										

DR, dietary record.

## DISCUSSION

We established an acrylamide database to calculate acrylamide intake for Japanese people and found that 17% of food items in the DRs and 19% of food items in the FFQ were designated as acrylamide-containing foods in the FCT. The deattenuated CC between 28-day DRs and the FFQ for validation was 0.37 to 0.54, weighted kappa coefficients were over 0.80, and energy-adjusted CC between FFQs for reproducibility was 0.56 to 0.62 in both sexes and both cohorts. The main contributing food group based on DR data was beverages, followed by confectioneries, vegetables, potatoes and starches, and cereals.

Our study showed a low-to-moderate correlation between DRs and the FFQ, and kappa coefficients indicated a high degree of coincidence. Moreover, correlations between the two FFQs were moderate. Therefore, acrylamide intake estimated from the FFQ is suitable for analysis as categorical variables in epidemiological studies, particularly for the JPHC prospective cohort study. The European Prospective Investigation into Cancer and Nutrition (EPIC) study reported a crude correlation of 0.35 between DRs and their FFQ.^20^ Our results are consistent with this previous report.

The mean intake and distribution in our study were similar to the results from the previous Monte Carlo simulation in Japan (mean, 0.166 µg/kg body weight/day; 95th percentile, 0.261 µg/kg body weight/day).^7^ The percentage difference between DRs and the FFQ in our study was about ±10%, and the distribution was also similar. Hence, we concluded that estimation of dietary acrylamide intake from DRs and the FFQ was relatively accurate. On the other hand, the estimated dietary acrylamide intake from Nagata et al’s FFQ is almost three times higher than our estimate.^21^ This difference may be partly due to differences in constructing the acrylamide database and calculating acrylamide intake. For example, meat products accounted for 12.4% of the total acrylamide intake in Nagata et al’s report,^21^ while no meat-based food items were designated as acrylamide-containing foods and the meat products group accounted for 0% of acrylamide intake in our study. In another example, in contrast to WHO’s report of a measured value for crumbed or battered poultry,^19^ we preferentially used a value for fried batter measured in Japan^7^ and did not use the value for crumbed or battered poultry to avoid double assessment of acrylamide intake from fried batter. Moreover, we categorized acrylamide intake from fried batter as cereals. These methodological differences may account for the differences in the contribution of the meat group and cereal food group to acrylamide intake. Therefore, differences in mean acrylamide intake levels among studies may be attributed to differences in study criteria and calculation methods used.

Our results indicate that acrylamide intake by Japanese people is about one tenth of that estimated by JECFA (1 µg/kg body weight/day for the general population).^22^ Moreover, the acrylamide intake of Japanese people is about a quarter of that of the Dutch population (0.45 µg/kg body weight/day),^5^ which corresponds to the amount of acrylamide derived only from potato crisps among the Dutch. These results suggest that acrylamide intake by Japanese people is much lower than that of western populations. However, a previous study reported that the margin of exposure is less than 10,000 when the benchmark dose lower confidence limit is 0.31 mg/kg body weight/day for mammary tumors in rats.^23^ Therefore, the Food Safety Commission of Japan calls for vigilance regarding the possibility of a carcinogenic effect of dietary acrylamide.^23^ Further, the results of epidemiological studies from western countries are inconsistent.^24^ Hence, epidemiological studies should be conducted to clarify the risk of dietary acrylamide intake on carcinogenicity in the Japanese population.

We found that acrylamide intake from vegetables accounted for a proportion of the total acrylamide intake, a finding which is consistent with previous reports in Japan.^7^^,^^21^ Contributing foods in western countries are primarily potato-based foods (eg, fried potato), wheat-based products (eg, biscuits), and coffee, but not vegetables.^25^^–^^27^ Therefore, cooked vegetables are an important source of acrylamide intake, at least in the Japanese population.

In our study, coffee, green tea, and vegetables accounted for about half of the total acrylamide intake. These foods are often reported to have preventive effects on cancers.^28^^–^^33^ Hence, an association analysis between acrylamide intake and cancers in the Japanese population is warranted and is likely to differ from the results from western countries.

The main strength of this study was the use of 28-day weighted DRs to calculate acrylamide intake. The use of 28-day records allowed us to better estimate the association between acrylamide intake and contributing foods from DRs than previous studies.

There are several limitations of this study. First, acrylamide intake from DRs and the FFQ may have been underestimated because the list of acrylamide-containing foods covered only about 17–19% of food items from each FCT. However, we considered home cooking methods for specific foods (mostly vegetables) and found that the contribution of vegetables was relatively high. This result indicates that cooking methods are important for estimating acrylamide intake. Second, we could not validate the calculated acrylamide intake from DRs. As acrylamide concentration varies even within the same food product depending on the cooking temperature and time,^3^ we used median or mean values of measurements to reduce the effect of such variations. Therefore, a validation study using duplicate methods is needed to clarify the accuracy of calculating acrylamide intake from DRs. Third, we could not compare dietary acrylamide intake and biomarkers, such as hemoglobin adduct concentrations of acrylamide and glycidamide. The Nurses’ Health Study II reported a moderate correlation between dietary acrylamide intake and hemoglobin adduct concentrations.^34^ However, dietary acrylamide levels are affected by individuals’ metabolism^35^ and lifestyle,^36^ causing sensitivity to dietary acrylamide to vary. This was reflected in the EPIC study, where, although acrylamide intake from the FFQ was correlated with that from DRs, the correlation between acrylamide intake from the FFQ and hemoglobin adduct concentrations was low.^20^ Therefore, analysis using biomarkers is needed to understand dietary acrylamide exposure in Japanese people.

The validity of estimating acrylamide intake using a FFQ was low to moderate in this study. However, the high validity of categorization suggests that the FFQ is suitable for use in epidemiological studies, particularly for the JPHC prospective cohort study. Further, the contributing foods to total acrylamide intake in the Japanese population were different from those in western countries, possibly due to differences in home food preparation methods. Therefore, considering household cooking methods, especially for vegetables, is important for assessing the acrylamide intake level in Japanese people.
